# Superior Mesenteric Artery Syndrome: A Report of a Rare Case

**DOI:** 10.7759/cureus.63572

**Published:** 2024-07-01

**Authors:** Sudhir Jayakar, Smitha Mogekar

**Affiliations:** 1 General Surgery, Dr. D. Y. Patil Medical College, Hospital and Research Centre, Dr D. Y. Patil Vidyapeeth, Pune, IND

**Keywords:** intestinal obstruction, mesenteric root syndrome, duodenal compression, aortomesenteric duodenal compression syndrome, aorto-mesenteric compass syndrome, cast syndrome, sma syndrome, superior mesenteric artery syndrome, superior mesenteric artery (sma), wilkie's syndrome

## Abstract

Superior mesenteric artery (SMA) syndrome is a rare disease in which the third part of the duodenum between the SMA and the abdominal aorta is compressed, leading to small bowel obstruction. Treatment is usually conservative, such as parenteral and nasojejunal nutrition. The pathophysiology includes loss of the retroperitoneal fat layer and subsequent duodenal compression. We present a 53-year-old malnourished female patient who came with complaints of vomiting, constipation, abdominal pain, and distension for four days. This article highlights the diagnostic challenges associated with SMA syndrome and emphasizes the importance of early diagnosis and intervention.

## Introduction

Superior mesenteric artery (SMA) syndrome is a very rare disease with an incidence of 0.1-0.3% [1]. It is more prevalent in females than males. It is characterized by the compression of the third part of the duodenum due to the reduced angle between the abdominal aorta and SMA [2]. Intestinal obstruction due to the compression of the third part of the duodenum by SMA and abdominal aorta is a controversial entity [3-6]. Not all patients experience serious gastrointestinal (GI) symptoms such as pain, vomiting, constipation, and abdominal distention; however, most patients experience the above symptoms over a long period of time [7]. Therefore, both clinical and radiological data are helpful in the diagnosis [1]. Delayed diagnosis can lead to many complications, including electrolyte imbalance, catabolic fatigue, gastric perforation, and peritonitis [8]. Conservative treatment has a place in managing such cases. Failure of conservative management warrants surgical intervention [1].

## Case presentation

A 53-year-old female homemaker presented to the emergency department with complaints of vomiting for four days, constipation for three days, and pain and distension in the upper abdomen for two days. The pain was insidious in onset and gradually progressive, especially in the upper abdomen and periumbilical area; the pain was crampy type and intermittent. The patient had vomited more than eight times a day, and the vomit contained food and bile, non-projectile. She had a history of pulmonary tuberculosis 20 years ago, had received anti-tuberculosis treatment (ATT) for six months, and had a history of significant weight loss during this period (not documented). The patient had an on-and-off history of similar complaints of pain in the abdomen, vomiting, and distension in the past and has taken symptomatic treatment at home. No other comorbidities were reported. Upon general physical examination, the patient was afebrile with a pulse rate of 100 per minute, blood pressure of 100/70 mmHg, respiratory rate of 20 cycles per minute, and body mass index (BMI) of 17.

**Figure 1:**
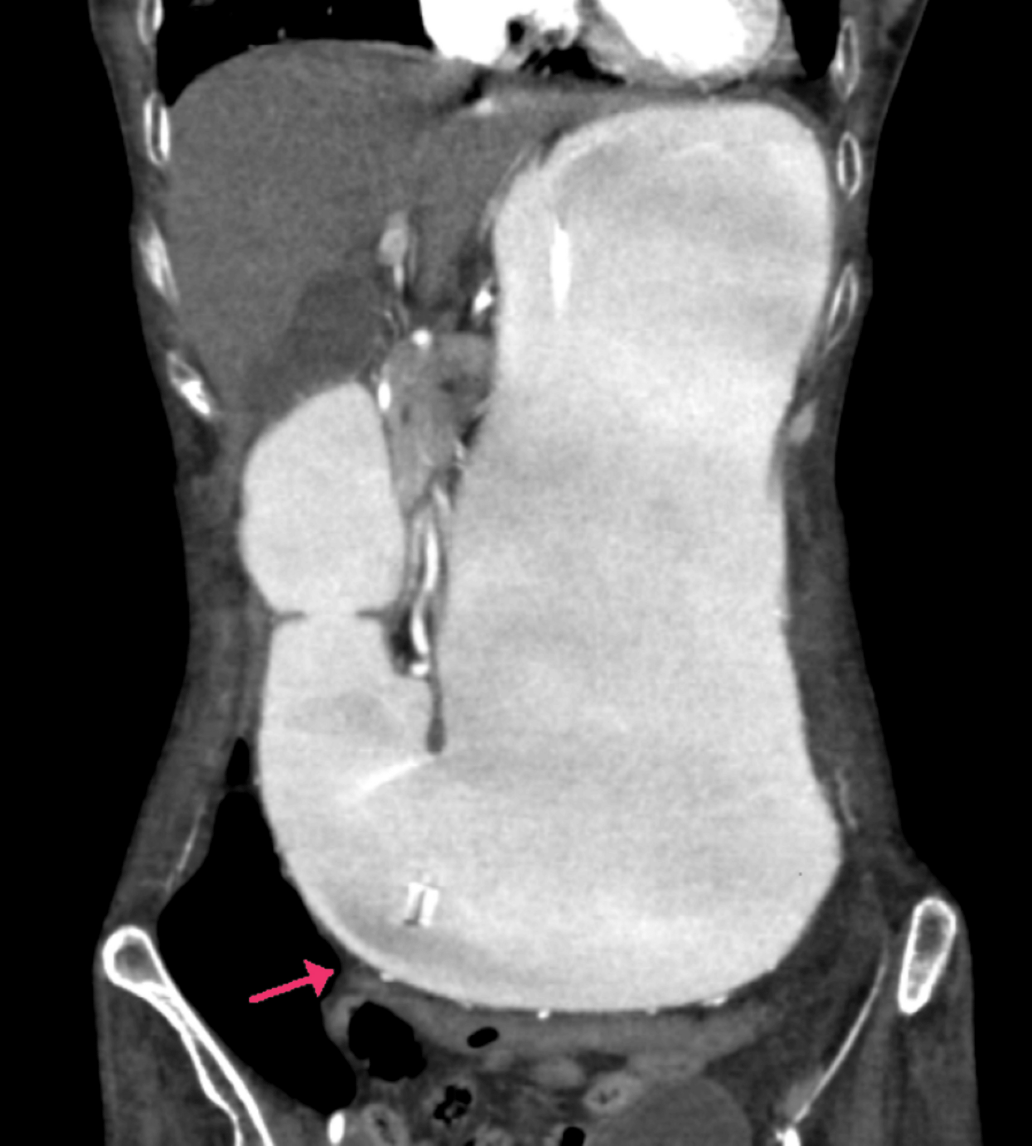
Contrast-enhanced computed tomography (CECT) of the abdomen revealed overdistention of the stomach with greater gastric curvature reaching toward the pelvic inlet

**Figure 2:**
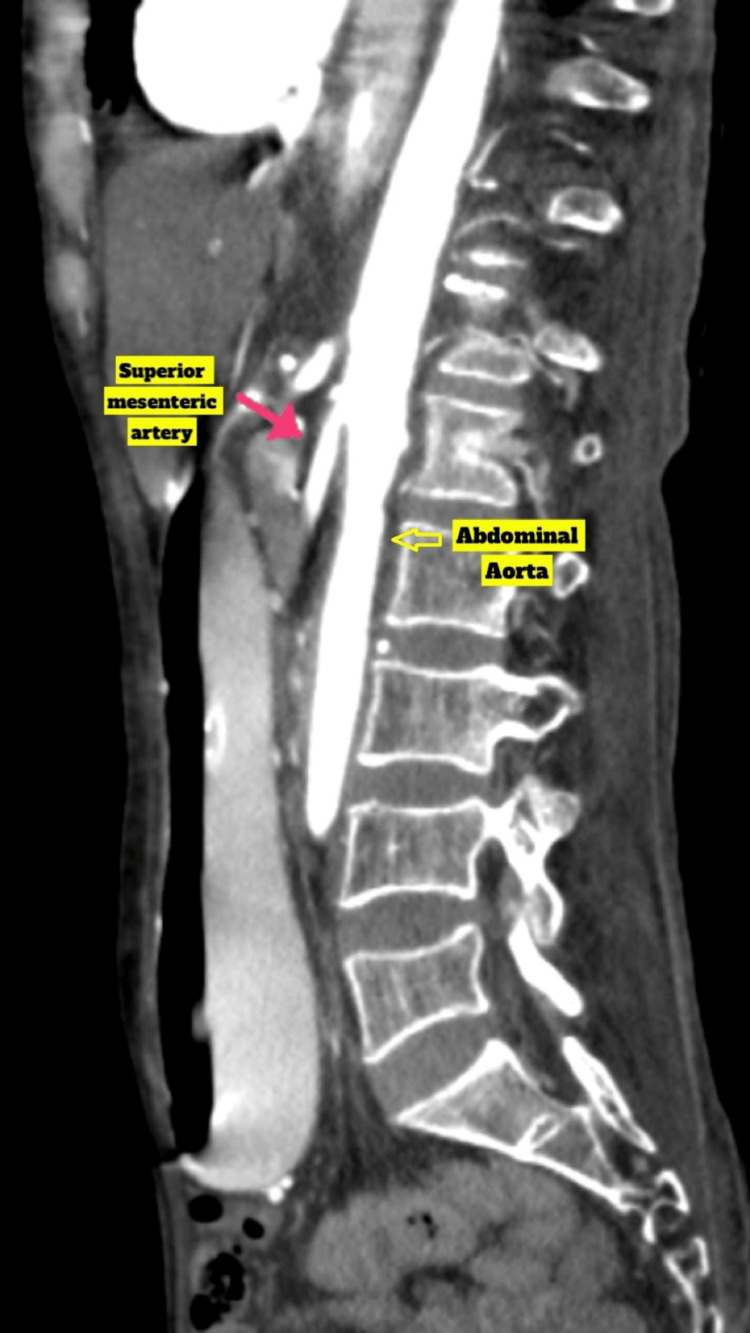
CT with angiography revealed decreased aorto-mesenteric angle and distance

During the abdomen examination, the abdomen was distended, with fullness noted in the epigastrium and peri-umbilical region. Tenderness was present in the epigastrium with no signs of guarding or rigidity. Bowel sounds were sluggishly present in all four quadrants. A digital rectal examination (DRE) revealed no stools. The patient was resuscitated with intravenous fluids according to the body weight along with anti-emetics. All routine blood investigations, such as complete blood picture, liver function tests, renal function tests, and serum electrolytes, were done and found to be within normal limits. However, serum protein revealed hypoalbuminemia. Ultrasound of the abdomen was suggestive of a distended stomach and sluggish peristalsis. Contrast-enhanced computed tomography (CECT) of the abdomen revealed overdistention of the stomach with greater curvature reaching up to the pelvic inlet (Figure 1). The first and second parts of the duodenum are also dilated, and narrowing of the third part of the duodenum is seen due to compression between the abdominal aorta and SMA with collapsed distal duodenum and jejunum loops. For further detailed evaluation, CT with angiography was done, which revealed the reduction in aorto-mesenteric angle (13°) and distance (4.5 mm) (Figure 2).

The patient was managed conservatively by keeping the patient nil by mouth for 72 hours due to obstruction, with input/output and abdominal girth charting. Nasogastric drainage was done to reduce the distension, and intravenous antibiotics, anti-emetics, and analgesics were given. For hypoalbuminemia correction, intravenous injection of Human Albumin 20% was given over four hours. Total parenteral nutrition was administered. Nasojejunal feeding was started after gastric decompression. Intestinal obstruction was relieved uneventfully (patient passed stools), and the patient was followed up for one year for definitive treatment. This case illustrates the clinical presentation, diagnostic workup, and successful conservative management of a patient with SMA syndrome presenting with significant GI symptoms and complications related to duodenal compression.

## Discussion

The SMA is a critical branch of the descending abdominal aorta, supplying the midgut from the second part of the duodenum to the proximal two-thirds of the transverse colon. The angle between the abdominal aorta and the origin of the SMA becomes significantly reduced in SMA syndrome, leading to duodenal compression. Typically, the angle between the abdominal aorta and the origin of the SMA is 25° to 60°, and the distance between the SMA and the abdominal aorta is 10 to 28 mm [1]. When these parameters decrease to approximately 6° to 16° and 2 to 8 mm, respectively, symptoms associated with SMA syndrome appear, such as abdominal pain, early satiety, nausea, vomiting, bloating, regurgitation, and abdominal distention, which usually appear later. The reduction of that angle may be due to any loss in the retroperitoneal pad of fat that leads to SMA syndrome [9,10]. Etiologically, SMA syndrome can be congenital, arising from anatomical abnormalities in the angle and distance between the SMA and abdominal aorta. Additionally, surgical or orthopedic factors that cause excessive weight loss, such as bariatric surgery, esophagectomy, or spinal manipulation, may also play a role. Other conditions that contribute to cachexia, such as malignancy, malabsorption syndrome, anorexia nervosa, HIV, and previous tuberculosis history, may also predispose people to SMA syndrome. For an accurate diagnosis, clinical suspicion confirmed through radiological investigations is important [8]. Initial testing usually includes an ultrasound in patients with abdominal pain to look for a distended stomach. Upper GI endoscopy helps exclude intraluminal causes and may reveal a dilated duodenum due to distal obstruction and reduced peristaltic waves characteristic of SMA syndrome. Barium studies showed gastric and duodenal dilatation due to slow gastroduodenal-jejunal transit. Similar studies, including CT or MR angiography, may help diagnose the disease by demonstrating vascular compression of the duodenum and measuring aorto-mesenteric angle and distance [11]. Management of SMA syndrome encompasses both medical and surgical interventions [12]. Conservative management focuses on gastric and duodenal decompression, correction of fluid and electrolyte imbalances, and nutritional support through total parenteral nutrition or nasojejunal nutrition. The goal of conservative management is to restore retroperitoneal fat and promote weight gain. Posturing maneuvers during meals and motility agents may be helpful in some patients [8,13]. If conservative treatment does not relieve the obstruction, surgery should be considered. Duodenojejunostomy is a surgery with a 90% success rate, which is the preferred surgical procedure [1]. Alternatively, procedures such as Strong's procedure [14] (including Treitz ligament division and duodenal mobilization) or gastrojejunostomy can be performed, but complications, such as blind loop syndrome and recurrence of symptoms, may occur after the surgery.

## Conclusions

SMA syndrome is rare but may be an important cause of intestinal obstruction. Clinician suspicion should be high, especially in patients with a persistent history of vomiting and weight loss. While upper GI endoscopy plays an important role in identifying other functional causes of duodenal obstruction, CECT provides detailed anatomical information to aid in the diagnosis of SMA syndrome. Raising awareness and early diagnosis are important to prevent unnecessary suffering. Early diagnosis allows timely intervention to improve patient outcomes and prevent complications associated with this rare but important clinical condition.
